# Emerging Trends of Infectious Diseases in Canada

**DOI:** 10.3390/tropicalmed11040102

**Published:** 2026-04-14

**Authors:** Yazdan Mirzanejad

**Affiliations:** Division of Infectious Diseases, Department of Medicine, Faculty of Medicine, University of British Columbia, Vancouver, BC V5Z 1M9, Canada; yazdan.mirzanejad@ubc.ca

As we conclude this set of ten publications in *Tropical Medicine and Infectious Disease*, three clear themes emerge to the surface. 

First, prevention succeeds when it is strategic and measurable. The HIV-focused contribution reminds us that pairing broad Treatment as Prevention—population-level viral suppression—with focused, high-impact PrEP delivery is not simply additive, but synergistic, provided programs are continuously monitored and adapted. 

Second, antimicrobial resistance is not merely theoretical—it is shaping real-world outbreaks and everyday prescribing practices. The multidrug-resistant Shigella outbreak among people experiencing homelessness underscores how transmission dynamics are inseparable from structural vulnerability and public health capacity. The Salmonella-focused work and the broader Canadian overview of Gram-negative resistance reinforce the practical value of regional susceptibility intelligence and stewardship-aligned empiric decisions. 

Third, diagnostics are increasingly integral to interventions. The mycobacterial sequencing papers demonstrate how clinically validated targeted sequencing can shorten time to actionable results—improving species identification for NTM disease and accelerating resistance detection for tuberculosis, with direct implications for patient outcomes. 

Finally, this collection encourages clinical humility: the case of the returning traveler highlights disciplined differential diagnosis, and the anti-IFN-γ autoantibody series reminds us that host immune factors can redefine what an “unusual infection” really means. Taken together, these papers convey a coherent message: prevention done well, resistance confronted with data, and diagnostics modernized for speed and precision—all grounded in patient care and public health realities. 

## Abbreviations

The following abbreviations are used in this manuscript:

PrEP	Pre-exposure Prophylaxis
NTM	Nontuberculous Mycobacteria
IFN-γ	Interferon Gamma

